# Altered Heart Rate Turbulence and Variability Parameters Predict 1-Year Mortality in Heart Failure with Preserved Ejection Fraction

**DOI:** 10.3390/jcdd9070213

**Published:** 2022-07-02

**Authors:** Jus Ksela, Lea Rupert, Anze Djordjevic, Miha Antonic, Viktor Avbelj, Borut Jug

**Affiliations:** 1 Department of Cardiovascular Surgery, University Medical Centre Ljubljana, 1000 Ljubljana, Slovenia; jus.ksela@kclj.si; 2 Faculty of Medicine, University of Ljubljana, 1000 Ljubljana, Slovenia; borut.jug@kclj.si; 3 Department of Anaesthesiology and Perioperative Intensive Therapy, University Medical Centre Ljubljana, 1000 Ljubljana, Slovenia; learupert@gmail.com; 4 Department of Cardiac Surgery, University Medical Centre Maribor, 2000 Maribor, Slovenia; anze.djordjevic@gmail.com; 5 Faculty of Medicine, University of Maribor, 2000 Maribor, Slovenia; 6 Department of Communication Systems, Jozef Stefan Institute, 1000 Ljubljana, Slovenia; viktor.avbelj@ijs.si; 7 Department of Vascular Diseases, University Medical Centre Ljubljana, 1000 Ljubljana, Slovenia

**Keywords:** heart failure with preserved ejection fraction, heart rate turbulence, heart rate variability, premature ventricular complex, mortality predictor

## Abstract

Background: Heart failure with preserved ejection fraction (HFpEF) is a complex and heterogeneous clinical syndrome. In the absence of effective and potent treatment strategies, the main challenge in HFpEF management remains the availability of strong predictors of unfavourable outcomes. In our study, we sought to evaluate the potential prognostic value of heart rate turbulence (HRT) and variability (HRV) parameters on mortality in ambulatory HFpEF patients. Methods: This was a case–control study comparing HRT and HRV parameters in HFpEF survivors vs. non-survivors. Patients from the RESPOND Heart Failure Registry with HFpEF who underwent 24 h ECG monitoring (Holter) were included; HRT parameters (i.e., turbulence onset (TO) and turbulence slope (TS)) and HRV parameters (i.e., standard deviation of NN intervals (SDNN)) derived from 24 h Holter ECGs were calculated in patients who died within 12 months, and compared to their age-, gender-, LVEF-, ECHO-, aetiology-, and therapy-matched alive controls. Results: A total of 22 patients (mean age 80 ± 7 years, 18% female, mean LVEF 57 ± 9%) were included in the final analysis. In deceased patients, values of TO were significantly higher, and values of TS and SDNN were significantly lower as compared to survivors. Conclusions: HRT and HRV parameters have the ability to differentiate individuals with HFpEF who are at the greatest risk of unfavourable outcomes. The extent of autonomic disbalance as determined by HRT and HRV could potentially assist in the prognostic assessment and risk stratification of HFpEF patients.

## 1. Introduction

Heart failure (HF) with preserved ejection fraction (HFpEF) is a complex and heterogeneous clinical syndrome affecting around 50% of all HF patients [1] and increasing in prevalence by about 1% annually, predominantly owing to the changing age, demographics and increasing prevalence of obesity in western societies [2]. It is characterised by significant morbidity and extremely grim prognosis, with a 5-year mortality rate reaching over 75% in most affected individuals [3,4,5]. With epidemic proportions of its incidence and without a single treatment modality consistently improving prognosis in these individuals, HFpEF remains one of the largest unmet clinical needs in 21st-century cardiovascular medicine [2,3].

With emerging new and revolutionary treatment strategies for the HFpEF patient population, prognostication of disease progression and patient deterioration is becoming increasingly important in order to build reliable prognostic models that would allow medical professionals and patients to develop realistic expectations about disease prognosis and choose the most appropriate monitoring and therapeutic strategies [3,4]. In recent years, evidence has accumulated that, aside from well-established HF-related clinical predictors, such as New York Heart Association (NYHA) class, left ventricular ejection fraction (LVEF), systolic blood pressure, QRS complex duration, atrial fibrillation (AF), or N-terminal-pro hormone B-type natriuretic peptide (NT-proBNP) [2], specific subclinical parameters, closely related to distinctive pathophysiological derangements observed in HFpEF, can provide crucial insight into the disease progression and, therefore, can be pivotal in tackling the HFpEF burden [1,6,7]. 

The pathophysiology of HF is characterised by profound haemodynamic abnormalities that also result in autonomic nervous system imbalance with an increase in sympathetic activity and withdrawal of vagal modulation [8,9,10,11]. Although significant sympathicovagal disbalance is crucial in the disease development and progression in virtually all HF patients, well-established conventional invasive and non-invasive techniques describing autonomic balance (such as heart rate variability, i.e., HRV), have shown limited ability to differentiate patients at greater risk for disease progression and/or unfavourable outcome [8,9,10].

In the last decade, heart rate turbulence (HRT), a novel marker of autonomic tone, representing the baroreflex-mediated short-term oscillation of cardiac cycle lengths after spontaneous premature ventricular complexes (PVCs) [12], has established itself as a reliable and powerful predictor of morbidity and mortality in various cardiovascular pathologies, such as myocardial infarction, lethal cardiac arrhythmias, or atrial fibrillation following open-heart surgery [12,13,14]. Whether HRT could potentially serve as a predictor of disease progression or dismal outcome also in HFpEF patients remains elusive.

The aim of the present study was to evaluate the potential prognostic value of HRT and HRV parameters on mortality in ambulatory HFpEF patients.

## 2. Materials and Methods

### 2.1. Patient Population 

A total of 723 patients with symptoms of HF from the RESPOND Heart Failure Registry [15] with 24 h Holter electrocardiography (ECG) monitoring recorded between 2014 and 2016 at the University Medical Centre Ljubljana were enrolled in this study. The National Ethics Committee approved the study protocol (protocol number 101/02/14), and the study was conducted according to the World Medical Association Declaration of Helsinki. All patients gave written informed consent prior to the enrolment.

Patients were included if they met the following criteria: (i) signs and symptoms of HF at the time of Holter monitoring recording [16]; (ii) echocardiographic evidence of preserved LVEF (LVEF ≥ 50%) [16] and structural cardiac abnormalities (hypertrophy, diastolic dysfunction, left atrial enlargement, and/or increased tricuspid regurgitation velocity [2]; (iii) N-terminal B-type natriuretic peptide (NT-proBNP) level > 600 pg/mL; (iv) preserved sinus rhythm; and (v) stable disease for at least 3 months prior to Holter monitoring recording [2,16].

Exclusion criteria included (i) an acute cardiovascular event within the past 3 months (e.g., myocardial infarction, stroke, or thromboembolic event) [16]; (ii) atrial fibrillation or other arrhythmias incompatible with HRT and/or HRV determination; and (iii) significant non-cardiac comorbidities with expected survival < 1 year [16]. 

### 2.2. Study Design

All patients underwent clinical examination, echocardiographic assessment, and 24 h Holter ECG recording. The documentation of all patients was retrospectively reviewed. Ischemic aetiology was defined as the presence of an angiographically proven obstructive atherosclerotic lesion ≥ 50% of at least one subepicardial coronary artery [16]. Hypertensive aetiology was defined as systolic pressure ≥ 140 mmHg or diastolic pressure ≥ 90 mmHg, history of arterial hypertension, and/or long-lasting antihypertensive therapy [16]. 

All patients were followed on an outpatient basis at the Heart Failure Clinic of the Department of Vascular Diseases, University Medical Centre Ljubljana, and evaluated by a dedicated cardiologist for a minimum of 12 months at regular, interval visits, as per the clinical pathway. If the patient missed a follow-up appointment, telephone contact with him/her, his/her relatives, or the general practitioner was carried out, and all relevant medical records were examined in order to assess any changes in the patient’s health status [16]. The primary outcome in our study was HF-related death (pump failure or sudden cardiac death) within 12 months after inclusion in the study. In all mortality cases, two additional independent cardiologists, blinded for baseline measurements, reconfirmed the observed endpoint [16].

### 2.3. HRT Analysis 

HRT has been proposed as a promising novel, non-invasive tool for risk stratification in patients suffering from various pathologies, including several distinctive cardiac diseases, such as myocardial infarction, lethal cardiac arrhythmias, or atrial fibrillation following open-heart surgery [12,13,14]. HRT describes short-term fluctuation in sinus cycle length that follows a PVC and basically describes how quickly and vigorously the heart reacts in response to a single premature ventricular complex [12]. Abnormal HRT reflects autonomic dysfunction, and a vast amount of literature indicated its potential ability in predicting cardiac death, sudden death, and all-cause mortality [12,13,14]. Its proven clinical significance lies mainly in its capability to predict mortality and sudden cardiac death following myocardial infarction, although some reports suggest that it is also applicable to many other cardiac pathologies (such as lethal cardiac arrhythmias or atrial fibrillation following open-heart surgery) [12,13,14] and non-cardiac diseases (such as liver failure or polycystic ovary syndrome) [17,18,19].

In this study, HRT parameters were calculated in a standardised fashion, as described in detail by Schmidt et al., using a dedicated and validated software system available from the currently discontinued web page popularising the non-commercial use of HRT (www.h-r-t.org, accessed by our research group firstly on 30 September 2009) [20]. In HRT analysis, 2 numerical descriptors were estimated: turbulence onset (TO), reflecting the amount of initial sinus rhythm acceleration following a PVC, and turbulence slope (TS), reflecting the rate of sinus rhythm deceleration that follows the initial sinus acceleration [12,20].

TO, expressed as a percentage, was calculated using the formula [(RR_1_ + RR_2_) − (RR_−2_ + RR_−1_)]/(RR_−2_ + RR_−1_) × 100, where RR_1_ and RR_2_ are the first and the second sinus RR intervals after a PVC and RR_−1_, and RR_−2_ are the first and the second sinus RR intervals preceding a PVC [12,20,21].

TS, expressed in ms/RR, was computed as a maximum positive slope of a regression line assessed over any of 5 consecutive RR intervals within the first 20 sinus RR intervals following a PVC [12,20,21].

While TO was computed for all suitable PVC in a Holter recording separately and then averaged, TS was calculated based on an average local tachogram [12,13,14,20]. Values of TO ≥ 0% and TS ≤ 2.5 ms/RR were used as cut-offs to consider HRT parameters abnormal [12,20].

Importantly, HRT analysis has several limitations, especially when the methodology is not depicted accurately [20,21,22,23,24]. For this reason, all our patients included in the final analysis met all inclusion criteria, and no exclusion criteria regarding the strict HRT methodology were set by the primary authors [20]. To summarise, to accurately calculate HRT parameters, 24 h Holter recordings were used with a minimum of 5 PVCs without prolonging recording time and excluding all interpolated PVCs. In order to cancel the dependency of HRT on heart rate, TS was adjusted to heart rate and the number of PVCs. Filtering criteria to ensure the utmost PVC quality were used according to Grimm et al. [22], and only PVC fulfilling all the following criteria were included in the final analysis: (i) index PVC embedded into at least five preceding and 20 succeeding normal RR intervals; (ii) cycle length of all considered RR intervals >300 ms but <2000 ms; (iii) beat-to-beat differences < 200 ms; and (iv) differences < 20% from the average of five preceding intervals [20,23,24].

### 2.4. HRV Analysis

Conventional, linear HRV analysis is a well-recognised non-invasive tool in studying cardiac autonomous modulation, exhibiting its usefulness in the diagnosis, characterisation, and classification of several cardiac pathologies, and providing information about the individual risk for adverse effects such as malignant rhythm disturbances or sudden cardiac death [8,25].

Among several linear HRV parameters, SDNN (standard deviation of the time interval between consecutive normal R waves from sinus beats) is the simplest and most widely used HRV parameter calculated from long-term ECG recordings, reflecting all long-term components responsible for the variability of the heart rate, including circadian rhythm and physical activity. A vast number of studies in the literature have proven SDNN as the most powerful predictor of HF-related death in progressive heart failure unrelated to underlying pathophysiology [26,27,28]. Furthermore, several commercially available devices which enable HRV measurement for sports professionals, recreational sportsmen, and the general population, calculate the HRV using SDNN as the sole and most reliable index of individuals’ HRV status [29].

Although it is known that HRV parameters are severely deranged in HF individuals [8,9,10,11], its usefulness in risk stratification in the HFpEF population is limited [25]. Identification of preselected linear, time-domain HRV parameters in our study was performed to objectively describe autonomic nervous status in our cohort of patients.

SDNN, mainly reflecting vagal modulation of autonomic balance, was calculated from 24 h Holter ECGs monitoring according to the Task Force of the European Society of Cardiology and the North American Society of Pacing and Electrophysiology’s recommendations using commercially available HolCard 24W analyser (Aspel, Poland) [25]. Standard automatic R wave peak detection algorithm was utilised as described in detail elsewhere [8,11,25]. Later, all recordings were manually reviewed and corrected, if necessary.

### 2.5. Statistical Analysis

Demographic and ECG-derived data of the two study groups were compared with the independent samples t-test for normally distributed variables, the Mann–Whitney U test for non-normally distributed variables, and the χ2 test for categorical variables, whereby the Kolmogorov–Smirnov test served as a normality check. A significant difference was considered when a p-value was less than 0.05. All statistical analyses were performed using the SPSS software package (IBM SPSS Statistics, version 20, Armonk, NY USA).

## 3. Results

### 3.1. Study Population

**Figure 1:**
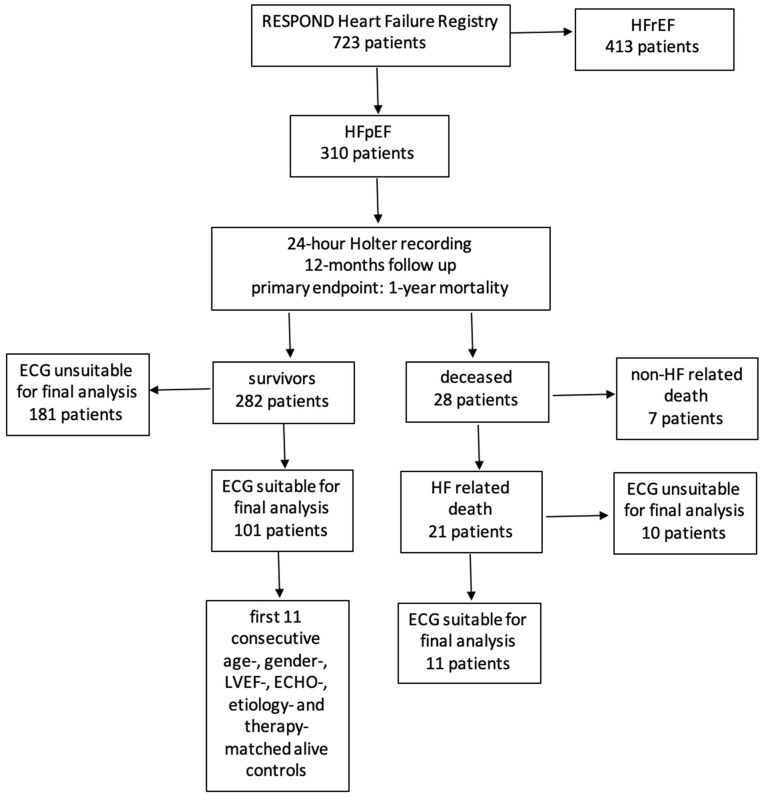
Patient flow diagram.

From a cohort of 723 HF patients undergoing 24 h ECG (Holter) monitoring, 310 patients with HFpEF and preserved sinus rhythm were identified. Of these, 282 patients survived the observed period, and 28 patients died within the first 12 months. Of the 28 deceased patients, 21 died due to HF-related causes, of whom 11 were suitable for further HRT and HRV analysis; from the 282 surviving patients, the recordings of 101 were deemed appropriate for further HRT and HRV analysis. In the end, 22 patients were selected: 11 cases (HFpEF who died) and the first consecutive 11 patients alive, used as age-, gender-, LVEF-, ECHO-, aetiology-, and therapy-matched controls (Figure 1).

The mean age of our population was 80 ± 7 years, 18% of all patients were female. Overall, 45% of our HFpEF patients were ischemic. Mean LVEF was 57 ± 9, and 55% of our research population were using beta-blockers, 73% RAAS inhibitors, 32% diuretics, and 23% mineralocorticoid receptor antagonists (Table 1). 

Although novel disease-modifying treatment strategies for HFpEF patients are currently promisingly evolving, the majority of affected individuals still receive beta-blockers—either to address co-morbidities (e.g., arterial hypertension) and pathophysiology (e.g., LV diastolic filling) or by adopting the same therapeutic approach to HFrEF and HFpEF patient populations. In fact, 75 % of patients with HFpEF still receive beta-blocking therapy [30,31]. Not only that beta-blockers address arterial hypertension, which is highly prevalent in this patient population [2], but several small-scale studies also indicate improved diastolic haemodynamic with heart-rate-reducing therapies [32]. Hence, more than half of our patient population was receiving beta-blocking therapy, which is in line with data from large-scale real-life registries of patients with HFpEF [33]. 

Additionally, while the majority of HFpEF patients are treated with diuretics, s significant number of patients in our cohort were in NYHA classes I and II at the time of Holter recordings and data acquisition; thus, diuretics were obviously discontinued for the time being in all compensated individuals.

### 3.2. HRT and HRV Values in Case–Control Population

**Figure 2:**
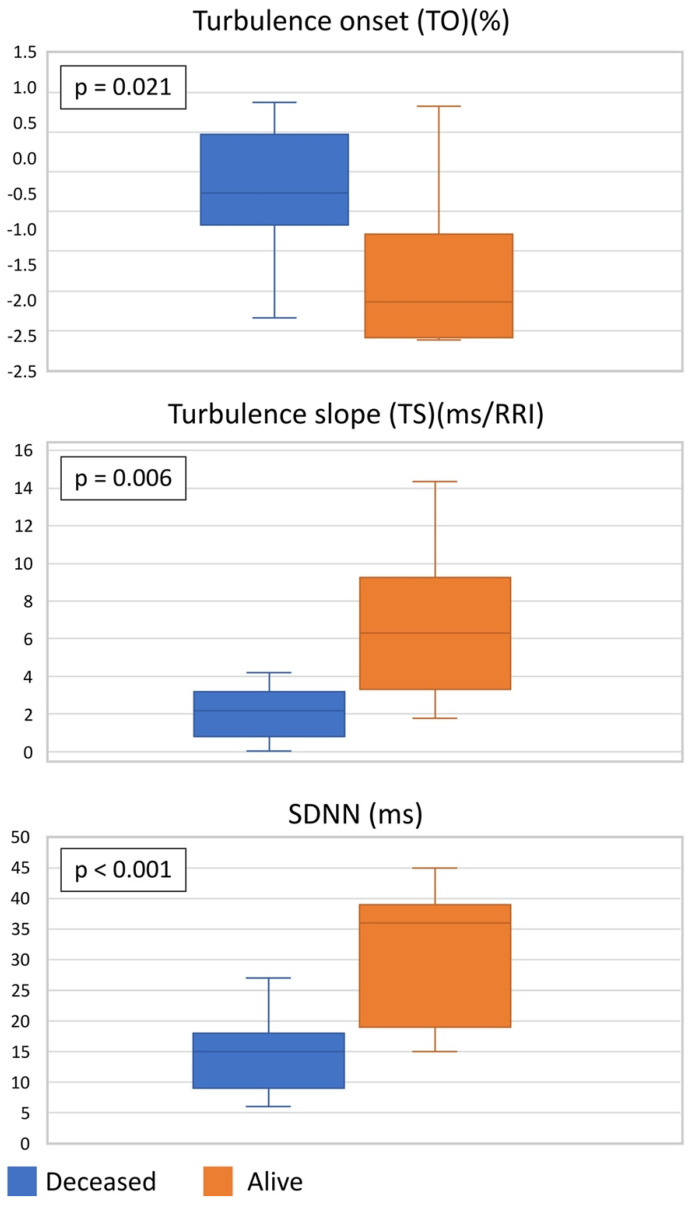
Summary of results. RRI: interval between two consecutive RR intervals; ms: millisecond; other abbreviations as in Table 2.

In deceased patients, TO was significantly higher (–0.27 [IQR –0.54 to 0.34] vs. –1.64 [IQR –1.99 to –1.01], *p* = 0.021), TS significantly lower (2.17 [IQR 0.80 to 3.08] vs. 6.29 [IQR 4.20 to 8.02], *p* = 0.006), and SDNN significantly lower (14.73 ± 6.97 vs. 30.55 ± 11.15 ms, *p* < 0.001) than in survivors, as seen in Table 2 and Figure 2.

## 4. Discussion

The main finding of our study is that deceased HFpEF patients had significantly higher values of TO and lower values of TS than their age-, gender-, LVEF-, ECHO-, aetiology-, and therapy-matched alive controls. Our results suggest that (i) HRT parameters may differentiate individuals with HFpEF at the greatest risk of unfavourable outcomes, and (ii) the extent of autonomic disbalance as determined by HRT could potentially assist in the prognostic assessment and risk stratification of HFpEF patients, which, in turn, may help us identify patients who would benefit the most from stringent clinical monitoring and intensified management strategies. 

Chronic HF is a life-threatening clinical syndrome with substantial morbidity and mortality [1,4], affecting 1–3% of the adult population and rapidly becoming one of the most prominent public health problems in industrial communities [2]. With the persistently rising prevalence and incidence [3], it currently represents one of the greatest and ever-growing burdens for patients, their families or carers, and the national healthcare systems. 

Among various subcategories of HF, HFpEF—a clinical entity characterised by symptoms of HF despite preserved LVEF—represents a particularly far-reaching medical challenge within the HF epidemics [1,4,5,34]. Currently, HFpEF affects approximately 5% of the general western population aged over 60 years [2,3] and represents nearly 50% of all HF cases [1]; it consumes almost half of all HF-related healthcare costs [5] and rises in prevalence by approximately 1% annually mainly due to increasing age and prevalence of hypertension, obesity, metabolic syndrome, and diabetes mellitus [3,4,5]. With limited effective treatment identified so far in pivotal clinical trials [4], HFpEF carries a particularly grim prognosis, with significant morbidity and mortality varying from 10% at 1 year to over 75% at 5 years in most affected individuals [3,4,5,35].

Limited therapeutic options in HFpEF are largely related to the complexity of the pathophysiology of the disease itself [34,35,36,37]. HFpEF, historically considered as being caused exclusively by left ventricular diastolic dysfunction [36], is a heterogeneous syndrome, caused by a complex interplay of multiple impairments in ventricular diastolic and systolic reserve, atrial function, systemic and pulmonary vascular function, nitric oxide bioavailability, chronotropic reserve, right heart function, autonomic tone, and peripheral impairments [1,4,34,35,36,37]. Multiple individual pathophysiological mechanisms, including cardiomyocyte stiffness, extracellular matrix remodelling, mitochondrial impairment, autophagy dysregulation, oxidative metabolic shift, calcium abnormalities, and alteration in electrical potential frequently coexist within the same patient to cause symptomatic HF, but between patients within the HFpEF population, the extent to which each component is operative can differ widely, confounding prognosis appraisal as well as monitoring and treatment approaches [3,4,5,9,35,36,37].

A vast number of studies in the literature indicate that neurohumoral activation, arising primarily as a compensatory mechanism in order to adjust cardiac performance in the face of increased workload and resulting in sympathetic overdrive and concomitant withdrawal of vagal activity, represents a major contributor to HF pathophysiology, regardless of its aetiology [8,9,10,11,38,39]. While the role of adrenergic hyperactivity with increased sympathetic nerve discharge, progressive loss of rhythmical sympathetic oscillations, and concomitant parasympathetic diminishment is increasingly well-appreciated and delineated in HF with reduced EF (HFrEF) [8,10], the role of reflex mechanisms in sustaining adrenergic abnormalities is less well understood in HFpEF [39]. Although the evidence clearly shows that HFpEF patients also have a prominent autonomic dysfunction [40], little is known about the role of neural mechanisms that govern the amplitude or frequency of bursts of autonomic activity, the pattern of active fibre discharge, or the central pathways that affect sympathetic burst generation [37]. Significantly lower values of SDNN parameter, a strong linear HRV index of vagal modulation in both alive and deceased HFpEF patients in our study (as compared to HRV values in healthy individuals reported in previous works [11]), clearly show that a markedly impaired HRV can be seen in HFpEF individuals, which is in synchrony with previous studies on sympathicovagal disbalance in HF patients [8,9,10,11]. Since SDNN is a strong indicator of parasympathetic activity, our results suggest that autonomic derangements in HFpEF are at least partially accompanied by withdrawal of vagal activity and, thus, cannot be solely attributed to sympathetic overdrive. 

In recent decades, it has become evident that one of the main challenges in tackling the HF epidemic is the assessment of the disease prognosis in order to individually choose the most appropriate monitoring and therapeutic strategies for various HF patients [3,36]. However, since HF is an extremely heterogeneous syndrome, a vast number of novel studies in the literature indicate that phenotype-specific or pathophysiology-related subcategories of HF [3] should be addressed separately when prognosticating unfavourable outcomes in the affected population, in order to avoid the bias of observing diverse HF patients as a uniform patient population. Although the LVEF-based categorisation of HF is often criticised for leading to oversimplification of the complexity of pathophysiological mechanisms involved in the disease development and progression, the majority of current international guidelines classify patients as having HF with reduced (HFrEF, EF < 40%), mid-range (HFmrEF, EF 40–49%), or preserved (HFpEF, EF > 50%) ejection fraction [3], a criterion that was also strictly followed in our study. Observing the SDNN parameter in our cohort of HFpEF patients, we showed that this marker was significantly lower in deceased HFpEF patients as compared to their alive controls, indicating that HFpEF patients with a more pronounced decline of autonomic regulation have a worse prognosis and a higher probability to experience an unfavourable event. Furthermore, deceased HFpEF patients in our study had also significantly higher values of TO and lower values of TS, both parameters of HRT, which is considered to be an indirect measure and surrogate of baroreflex sensitivity. Since virtually no strong data exist on arterial baroreflex control mechanisms and autonomic dysfunction coupling in ‘true’ HFpEF (i.e., LVEF > 50%) [41,42,43,44,45,46], our results are especially interesting. Specifically, our study shows that HFpEF patients with abnormal baroreflex sensitivity or arterial baroreflex control have a worse prognosis and that they die before their age-, gender-, LVEF-, ECHO-, aetiology-, and medical therapy-matched controls. Findings that HRT parameters could differentiate between HFpEF patients with a greater risk of unfavourable outcome (i.e., 1-year morality) could potentially complement the prognostication process in order to build reliable prognostic models that would allow medical professionals and patients to develop realistic expectations about HFpEF prognosis and choose the most appropriate monitoring and therapeutic strategies in this delicate subcategory of the HF population.

Although our study has identified that HFpEF patients with markedly declined autonomic modulation and baroreceptor sensitivity as appraised by HRT analysis have a worse prognosis, some limitations of our work should be addressed. Firstly, and most importantly, our study included only 22 HFpEF patients, which is a relatively small number, diminishing the power of our study. However, since this is a pilot study observing HRT in HFpEF cohorts, we strongly believe that at least a subtle insight into the simpatico-vagal balance is achievable by observing small cohorts of HFpEF individuals. Secondly, during our research, we strictly included only HF patients with LVEF > 50%, which is completely in synchrony with current international guidelines; however, this fact makes our results harder to compare to previous studies of HRT derangement in HF patients, in which HFpEF was consistently defined as LVEF>30% or >35% [41,42,43,44,45,46]. Although strict adherence to guideline criteria is, in our opinion, a strength of our study rather than a limitation, this fact should be considered when our results are compared to previous studies on autonomic dysfunction in HFpEF cohorts. Thirdly, our study does not provide insight into pathophysiological pathways involved in baroreflex sensitivity and autonomic derangements coupling and the exact mechanisms of arterial baroreflex control in HFpEF patients; thus, further research addressing these issues is anticipated. 

## 5. Conclusions

With emerging new and revolutionary therapeutic options for the HFpEF patient population, prognostication of disease progression and patient deterioration is becoming increasingly important. Our study is the first to address the prognostic value of HRT and HRV in the ambulatory chronic ‘true’ HFpEF population as defined by current international guidelines (i.e., EF > 50%). We showed that markedly elevated values of TO and decreased values of TS and SDNN are associated with worse 1-year prognosis in ‘true’ HFpEF patients. Thus, our work indicates that HRT and HRV have the ability to differentiate individuals with HFpEF who are at the greatest risk of unfavourable outcomes and that the extent of autonomic disbalance as determined by HRT and HRV could potentially assist in the prognostic assessment and risk stratification of HFpEF patients and may potentially help identify patients who would most benefit from stringent monitoring and intensified treatment strategies. 

## Figures and Tables

**Table 1:** Baseline characteristics of the study population between deceased group of patients and alive control group patients.

Characteristic	All Patients (n = 22)	Deceased (n = 11)	Alive Controls (n = 11)	*p*-Value
Age (years)	80 ± 7	80 ± 6	80 ± 7	0.872
Gender, female (%)	4 (18)	2 (18)	2 (18)	1.000
NYHA class ≥ III (%)	6 (27)	3 (27)	3 (27)	1.000
Therapy				
ACEi/ARB (%)	16 (73)	8 (73)	8 (73)	1.000
Spironolactone (%)	5 (23)	3 (27)	2 (18)	0.611
Diuretics (%)	7 (32)	3 (27)	4 (36)	0.647
Beta-blockers (%)	12 (55)	6 (55)	6 (55)	1.000
LVEF				
Mean	63 ± 6	63 ± 2	64 ± 1	0.735
50–60% (%)	11 (50)	6 (55)	5 (45)	0.669
60–70% (%)	6 (27)	3 (27)	3 (27)	1.000
>70% (%)	5 (23)	2 (18)	3 (27)	0.611
HF Aetiology				
ischemic (%)	10 (45)	5 (45)	5 (45)	0.669
non-ischemic (%)	12 (55)	6 (55)	6 (55)	0.669
Diastolic Dysfunction				
Grade I (%)	12 (55)	6 (55)	6 (55)	1.000
Grade II (%)	8 (36)	4 (36)	4 (36)	1.000
Grade III (%)	2 (9)	1 (9)	1 (9)	1.000
LV Hypertrophy	15 (68)	8 (72)	7 (64)	0.476
LA Enlargement	13 (59)	7 (64)	6 (55)	0.603
E/A Ratio	0.8 ± 0.2	0.7 ± 0.3	0.8 ± 0.1	0.729
E/e’ Ratio	10 ± 4	10 ± 0.2	9 ± 3	0.579
TR Vmax	2.7 ± 0.6	2.6 ± 0.7	2.8 ± 0.4	0.638
Heart Rate (beats/minute)	79.6 ± 5.3	79.4 ± 4.9	80.1 ± 5.8	0.454

ACEi: angiotensin-converting enzyme inhibitor; ARB: angiotensin receptor blocker; LVEF: left ventricular ejection fraction; HF: heart failure; NYHA: New York Heart Association; LV: left ventricle; LA: left atrium; E: peak early mitral inflow velocity; A: peak velocity flow in late diastole; e′: peak early diastolic mitral annular velocity; TR Vmax: maximal tricuspid regurgitation velocity.

**Table 2:** Heart rate turbulence and time-domain heart rate variability analysis.

	Deceased (n = 11)	Alive Controls (n = 11)	*p*-Value
Number of PVCs (median, IQR)	17 [13–19]	14 [11–19]	n/a
TO (%) (median, IQR)	−0.27 [−0.54–0.34]	−1.64 [−1.99–−1.01]	0.021
TS (ms/RRI)	2.17 [0.80–3.08]	6.29 [4.20–8.02]	0.006
SDNN (ms)	14.73 ± 6.97	30.55 ± 11.15	<0.001

PVC: premature ventricular complex, TO: turbulence onset; TS: turbulence slope, IQR: interquartile range; SDNN standard deviation of NN intervals.

## Data Availability

The data used to support the findings of this study are available from the corresponding author upon request.

## References

[1] Louridas G.E., Lourida K.G. (2016). Heart failure in patients with preserved ejection fraction: Questions concerning clinical progression. J. Cardiovasc. Dev. Dis..

[2] McDonagh T.A., Metra M., Adamo M., Gardner R.S., Baumbach A., Böhm M., Burri H., Butler J., Celutkiene J., Chioncel O. (2021). 2021 ESC guidelines for the diagnosis and treatment of acute and chronic heart failure: Developed by the task force for the diagnosis and treatment of acute and chronic heart failure of the European Society of Cardiology (ESC) with the special contribution of the Heart Failure Association (HFA) of the ESC. Eur. Heart J..

[3] Chioncel O., Lainscak M., Seferovic P.M., Anker S.D., Crespo-Leiro M.G., Harjola V.P., Parissis J., Laroche C., Piepoli M.F., Fonseca C. (2017). Epidemiology and one-year outcomes in patients with chronic heart failure and preserved, mid-range and reduced ejection fraction: An analysis of the ESC Heart Failure Long-Term Registry. Eur. J. Heart Fail..

[4] Anker S.D., Butler J., Filippatos G., Ferreira J.P., Bocchi E., Böhm M., Brunner-La Rocca H.P., Choio D.J., Chopra V., Chuquiure-Valenzuela E. (2021). Empagliflozin in Heart Failure with a Preserved Ejection Fraction. N. Engl. J. Med..

[5] Liao L., Jollis J.G., Anstrom K.J., Whellan D.J., Kitzman D.W., Aurigemma G.P., Mark D.B., Schulman K.A., Gottdiener J.S. (2006). Costs for Heart Failure With Normal vs Reduced Ejection Fraction. Arch. Intern. Med..

[6] Savic-Radojevic A., Pljesa-Ercegovac M., Matic M., Simic D., Radovanovic S., Simic T. (2017). Novel biomarkers of heart failure. Adv. Clin. Chem..

[7] Borlaug B.A., Redfield M.M., Melenovsky V., Kane G.C., Karon B.L., Jacobsen S.J., Rodeheffer R.J. (2013). Longitudinal changes in left ventricular stiffness: A community-based study. Circ. Heart Fail..

[8] Ksela J., Avbelj V., Kalisnik J.M. (2015). Multifractality in heartbeat dynamics in patients undergoing beating-heart myocardial revascularization. Comput. Biol. Med..

[9] Berezin E.A., Berezin A.A., Lichtenauer M. (2021). Myokines and heart failure: Challenging role in adverse cardiac remodelling, mypothy, and clinical oputcomes. Dis. Markers.

[10] Liu L., Wu Q., Yan H., Chen B., Zheng X., Zhou Q. (2020). Association between Cardiac Autonomic Neuropathy and Coronary Artery Lesions in Patients with Type 2 Diabetes. Dis. Markers.

[11] Grässler B., Thielmann B., Böckelmann I., Hökelmann A. (2021). Effects of different exercise interventjons on cardiac autonomic control and secondary health factors in middle-aged adults: A systematic review. J. Cardiovasc. Dev. Dis..

[12] Cygankiewicz I. (2013). Heart rate turbulence. Prog. Cardiovasc. Dis..

[13] Pinnacchio G., Lanza G.A., Stazi A., Careri G., Coviello I., Mollo R., Crea F. (2015). Determinants of heart rate turbulence in individuals without apparent heart disease and in patients with stable coronary artery disease. Europace.

[14] Marynissen T., Flore V., Heidbuchel H., Nuyens D., Ector J., Willems R. (2014). Heart rate turbulence predicts ICD-resistant mortality in ischaemic heart disease. Europace.

[15] Traxler D., Lainscak M., Simader E., Ankersmit H.J., Jug B. (2017). Heat shock protein 27 acts as a predictor of prognosis in chronic heart failure patients. Clin. Chim. Acta.

[16] Kosir G., Jug B., Novakovic M., Bozic Mijovski M., Ksela J. (2019). Endocan Is an Independent Predictor of Heart Failure-Related Mortality and Hospitalizations in Patients with Chronic Stable Heart Failure. Dis. Markers.

[17] Jansen C., Al-Kassou B., Lehmann J., Pohlmann A., Chang J., Praktiknjo M., Nickenig G., Strassburg C.P., Schrickel J.W., Andrie R. (2018). Severe abnormal Heart Rate Turbulence Onset is associated with deterioration of liver cirrhosis. PLoS ONE.

[18] Özkeçeci G., Ünlü B.S., Dursun H., Akci Ö., Köken G., Onrat E., Avsar A. (2016). Heart rate variability and heart rate turbulence in patients with polycystic ovary syndrome. Anatol. J. Cardiol..

[19] Yu Y., Xu Y., Zhang M., Wang Y., Zou W., Gu Y. (2018). Value of Assessing Autonomic Nervous Function by Heart Rate Variability and Heart Rate Turbulence in Hypertensive Patients. Int. J. Hypertens..

[20] Schmidt G., Malik M., Barthel P., Schneider R., Ulm K., Rolnitzky L., Camm A.J., Bigger J.T., Schömig A. (1999). Heart-rate turbulence after ventricular premature beats as a predictor of mortality after acute myocardial infarction. Lancet.

[21] Bauer A., Malik M., Barthel P., Schneider R., Watanabe M.A., Camm A.J., Schömig A., Schmidt G. (2006). Turbulence dynamics: An independent predictor of late mortality after acute myocardial infarction. Int. J. Cardiol..

[22] Grimm W., Sharkova J., Christ M., Schneider R., Schmidt G., Maisch B. (2003). Heart Rate Turbulence following Ventricular Premature Beats in Healthy Controls. Ann. Noninvasive Electrocardiol..

[23] D’Addio G., Cesarelli M., Corbi G., Romano M., Furgi G., Ferrara N., Rengo F. (2010). Reproducibility of heart rate turbulence indexes in heart failure patients. Annu. Int. Conf. IEEE Eng. Med. Biol. Soc..

[24] Blesius V., Schölzel C., Ernst G., Dominik A. (2020). HRT assessment reviewed: A systematic review of heart rate turbulence methodology. Physiol. Meas..

[25] (1996). Heart rate variability: Standards of measurement, physiological interpretation and clinical use. Task Force of the European Society of Cardiology and the North American Society of Pacing and Electrophysiology. Circulation.

[26] Cygankiewicz I., Zareba W. (2013). Heart rate variability. Handb. Clin. Neurol..

[27] Xhyheri B., Manfrini O., Mazzolini M., Pizzi C., Bugiardini R. (2012). Heart rate variability today. Prog. Cardiovasc. Dis..

[28] Nolan J., Batin P.D., Andrews R., Lindsay S.J., Brooksby P., Mullen M., Baig W., Flapan A.D., Cowley A., Prescott R.J. (1998). Prospective study of heart rate variability and mortality in chronic heart failure: Results of the United Kingdom heart failure evaluation and assessment of risk trial (UK-heart). Circulation.

[29] Hernando D., Roca S., Sancho J., Alesanco A., Bailon R. (2018). Validation of the Apple Watch for Heart Rate Variability Measurements during Relax and Mental Stress in Healthy Subjects. Sensors.

[30] Solomon S.D., McMurray J.J.V., Anand I.S., Ge J., Lam C.S.P., Maggioni A.P., Martinez F., Packer M., Pfeffer M.A., Pieske B. (2019). Angiotensin-Neprilysin Inhibition in Heart Failure with Preserved Ejection Fraction. N. Engl. J. Med..

[31] Silverman D.N., Plante T.B., Infeld M., Callas P.W., Juraschek S.P., Dougherty G.B., Meyer M. (2019). Association of β-Blocker Use With Heart Failure Hospitalizations and Cardiovascular Disease Mortality Among Patients With Heart Failure With a Preserved Ejection Fraction: A Secondary Analysis of the TOPCAT Trial. JAMA Netw. Open.

[32] Gorre F., Vandekerckhove H. (2010). Beta-blockers: Focus on mechanism of action. Which beta-blocker, when and why?. Acta Cardiol..

[33] Crespo-Leiro M.G., Anker S.D., Maggioni A.P., Coats A.J., Filippatos G., Ruschitzka F., Ferrari R., Piepoli M.F., Delgado Jimenez J.F., Metra M. (2016). European Society of Cardiology Heart Failure Long-Term Registry (ESC-HF-LT): 1-year follow-up outcomes and differences across regions. Eur. J. Heart Fail..

[34] Badreldin H.A., Aldosari N., Alnashwan L., Almutairi T., Yousif N., Alsulaiman K., Aljuhani O., Hafiz A., Alshaya O. (2022). What the near future holds for sacubitril/valsartan: A summary of major ongoing studies. J. Cardiovasc. Dev. Dis..

[35] Harper A.R., Patel H.C., Lyon A.R. (2018). Heart failure with preserved ejection fraction. Clin. Med..

[36] Meta-analysis Global Group in Chronic Heart Failure (MAGGIC) (2012). The survival of patients with heart failure with preserved or reduced left ventricular ejection fraction: An individual patient data meta-analysis. Eur. Heart J..

[37] Nandini N. (2020). Epidemiology and pathogenesis of heart failure with preserved ejection fraction. Rev. Cardiovasc. Med..

[38] Toschi-Dias E., Rondon M.U.P.B., Cogliati C., Paolocci N., Tobaldini E., Montano N. (2017). Contribution of Autonomic Reflexes to the Hyperadrenergic State in Heart Failure. Front. Neurosci..

[39] Florea V.G., Cohn J.N. (2014). The Autonomic Nervous System and Heart Failure. Circ. Res..

[40] Filgueiras-Rama D., Zipes D.P., Jalife J., Stevenson W.G. (2018). Sympathetic Innervation and Cardiac Arrhythmias. Cardiac Electrophysiology: From Cell to Bedside.

[41] Barthel P., Schneider R., Bauer A., Ulm K., Schmitt C., Schömig A., Schmidt G. (2003). Risk stratification after acute myocardial infarction by heart rate turbulence. Circulation.

[42] Makikallio T.H., Barthel P., Schneider R., Bauer A., Tapanainen J.M., Tulppo M.P., Schmidt G., Huikuri H.V. (2005). Prediction of sudden cardiac death after acute myocardial infarction: Role of Holter monitoring in the modern treatment era. Eur. Heart J..

[43] Bauer A., Barthel P., Schneider R., Ulm K., Müller A., Joeining A., Stich R., Kiviniemi A., Hnatkova K., Huikuri H. (2009). Improved Stratification of Autonomic Regulation for risk prediction in post-infarction patients with preserved left ventricular function (ISAR-Risk). Eur. Heart J..

[44] Bauer A., Barthel P., Müller A., Ulm K., Huikuri H., Malik M., Schmidt G. (2009). Risk prediction by heart rate turbulence and deceleration capacity in postinfarction patients with preserved left ventricular function retrospective analysis of 4 independent trials. J. Electrocardiol..

[45] Cygankiewicz I., Zareba W., Vazquez R., Bayes-Genis A., Pascual D., Macaya C., Almendral J., Fiol M., Bardaji A., Gonzalez-Juanatey J.R. (2009). Risk stratification of mortality in patients with heart failure and left ventricular ejection fraction >35%. Am. J. Cardiol..

[46] La Rovere M.T., Pinna G.D., Maestri R., Barlera S., Bernardinangeli M., Veniani M., Nicolosi G.L., Marchioli R., Tavazzi L. (2012). Autonomic markers and cardiovascular and arrhythmic events in heart failure patients: Still a place in prognostication? Data from the GISSI-HF trial. Eur. J. Heart Fail..

